# High-Frequency Repetitive Transcranial Magnetic Stimulation Could Improve Impaired Working Memory Induced by Sleep Deprivation

**DOI:** 10.1155/2019/7030286

**Published:** 2019-12-12

**Authors:** Zhiwei Guo, Zhijun Jiang, Binghu Jiang, Morgan A. McClure, Qiwen Mu

**Affiliations:** ^1^Department of Radiology and Rehabilitation, Imaging Institute of Brain Function, The Second Clinical Medical College of North Sichuan Medical College, Nanchong Central Hospital, Nanchong, China 637000; ^2^Department of Radiology, Peking University, Third Hospital, Beijing, China

## Abstract

**Objective:**

To investigate whether and how the working memory impairment induced by sleep deprivation (SD) could be recovered by using repetitive transcranial magnetic stimulation (rTMS), as well as to clarify the corresponding brain activity changes.

**Methods:**

Seventeen healthy adults received one session of 5.0 Hz rTMS over the left dorsolateral prefrontal cortex (DLPFC) following 24 hours of SD. Resting state functional magnetic resonance imaging (fMRI) and working memory test were performed during a rested waking period, after SD and rTMS. The amplitude of low-frequency fluctuations (ALFF) was used to detect the spontaneous neural activity changes after both SD and rTMS. The relationship between ALFF and the performance of working memory was also assessed by using correlation analysis.

**Results:**

After SD, the participants exhibited lower response accuracies and longer reaction times on the working memory tests of letters and numbers. The decreased response accuracy of numbers was significantly improved after rTMS similarly to the state of the rested waking period after a normal night of sleep. ALFF values decreased from the rested waking period state to the state of SD in the brain regions involving the frontal gyrus, precuneus, angular gyrus, and parietal lobe which showed significantly increased ALFF after rTMS. Furthermore, significantly positive correlations were observed between changes of response accuracy and the changes of ALFF value of the inferior frontal gyrus and supramarginal gyrus.

**Conclusion:**

These results indicate that high-frequency rTMS applied over left DLPFC may contribute to the recovery of the impaired working memory after SD by modulating the neural activity of related brain regions.

## 1. Introduction

Substantial studies have reported that sleep deprivation (SD) impairs various aspects of cognition involving working memory, verbal learning, attention, and processing speed [1–5]. Fatigue and sleepiness induced by SD are always related to higher mistakes, declined work efficiency, and elevated risk of accidents [1, 6]. Long-term SD also negatively affects the metabolic, physiological, and psychological reactivity which may lead to serious somatic illnesses [7–9]. However, for some special professions, such as a soldier or a doctor, SD often exists. To elucidate the mechanism of SD and how to retrieve the accompanied cognitive ability is therefore very important for minimizing medical accident and military loss.

Previous neuroimaging studies that utilized functional magnetic resonance imaging (fMRI), arterial spin labeling (ASL), and positron emission tomography (PET) have suggested that detriments in specific cognitive functions accompanying SD can be associated with the abnormal changes of regional cerebral blood flow, metabolic rate of glucose, and neural activation, as well as the functional brain networks of cerebral systems [2, 10–13]. These influences on cerebral responses show hyperactivation or lower activation which were most prominent in cognition-related brain areas involving the prefrontal cortex, parietal lobe, cingulate gyrus, thalamus, angular gyrus, hippocampus, and cerebellum [2, 10–12, 14, 15]. In particular, the activation of the angular gyrus and thalamus was reported significantly associated with the deterioration of behavioral performances [12, 14].

Numerous investigations have proved that the aberrant spontaneous neural activity and functional networks of several diseases could be modulated by using repetitive transcranial magnetic stimulation (rTMS) through changing the excitability of the site of stimulation as well as the distant anatomically connected sites [16–22]. Previous rTMS studies on patients with cognition impairment in Alzheimer's disease, Parkinson's disease, schizophrenia, and dementia in older patients have reported significant positive effectiveness on improving cognitive ability [23–26]. Similar results were reported in both animal and human studies on SD which also observed the potential effectiveness of rTMS for the treatment of cognitive impairment induced by SD, especially the working memory [27–31]. One human study also observed the significant correlation between the cortical activation changes induced by SD and the enhanced working memory performance during rTMS over the identical cortical site. In this study, working memory task-related fMRI was performed before and at the end of SD to identify the cortical regions that show enhanced or decreased activation induced by SD. These regions were selected as the stimulation sites of rTMS. Based on these results, the authors suggested that rTMS administered to these sites related to SD might reverse or remediate deterioration in task performance [30], but no fMRI examination was conducted after rTMS, and working memory-associated brain activation changes after rTMS could not be observed. In another human study [31], rTMS sessions were performed concurrently with working memory task performance during the SD period, both before and after, in which the fMRI scan was arranged. After comparing with sham rTMS, increased activation during SD was observed in the left middle occipital gyrus which was directly beneath the center of the rTMS coil. Facilitation of working memory performance was also detected in the group receiving active rTMS. These possibly indicate the effect of rTMS on working memory performance and the corresponding neural activity.

However, the following questions needed to be addressed: First, the correlation between network activation changes induced by SD and enhanced working memory performance with rTMS could not directly validate the potential influence of rTMS on neural circuitry. No comparison of the neural activation after rTMS and SD was conducted which could reflect the effect of rTMS on neural circuitry directly. Second, task-fMRI was used in these previous studies, but little is known about how rTMS influences the spontaneous neural activity and modulates the cognition-related functional network after SD.

To resolve these issues, we recruited healthy young participants with the same educational background to investigate the alteration of working memory performance before and after 24-hour SD. The fMRI data of three statuses (after a normal night of sleep, after 24 hours of SD, and after rTMS stimulation) were collected and utilized to examine the brain activity changes. Amplitude of low-frequency fluctuations (ALFF) has the ability to locate the altered regional spontaneous brain activity and is a useful tool to explore various potential neurobiological mechanisms [32]. We hypothesized that rTMS could recover the changes of neural activities of cognition-related brain areas after SD which were associated with the reversion of working memory performance. ALFF was applied to distinguish different spontaneous neural activities of SD status and rTMS status.

## 2. Materials and Methods

### 2.1. Subjects

Seventeen right-handed undergraduate students aged from 21 to 26 (23.00 ± 1.37 years), 13 females and four males, were recruited in this study. All participants were healthy with no history of any neurological, psychiatric, cardiovascular, or sleep disorders. They met the following inclusion and exclusion criteria: (1) no alcohol, caffeine, morphine, or opioid intake within one week prior to the experiment; (2) 7-9 hours of habitual sleep schedules on average per night and no insomnia; and (3) an absence of TMS and MRI contraindications. During the study, smoking, caffeine, and alcohol intake were inhibited. Written informed consent was obtained from all participants according to the Declaration of Helsinki prior to enrollment into the study. This study was approved by the local ethics committee of the Second Clinical Medical College of North Sichuan Medical College.

### 2.2. Experiment Procedures

For each subject, the whole experiment period extended for three days. Subjects followed normal sleep schedules from 10:00 PM on the first day to 7:00 AM on the second day (a normal night of sleep). Sleep deprivation was carried out from the morning on the second day at 7:00 AM to the morning on the third day at 7:00 AM during which the subjects were arranged in a separate room and were constantly monitored (sleep deprivation). During the period of sleep deprivation, subjects were allowed to play games, watch videos, chat with each other, or do other nonstrenuous activities, but they were not allowed to rest with their eyes closed even for a short time. Also, caffeine, alcohol, and smoking were all prohibited during this period in any amount. After the sleep deprivation, a session of rTMS was provided to investigate the potential effects of rTMS on improving the cognitive function after SD.

### 2.3. rTMS Procedures

Each participant received a treatment course of rTMS applied over the left dorsolateral prefrontal cortex (DLPFC) after the 24 hours of sleep deprivation according to the following protocol: 20 trains of 50 pulses with a 50-second intertrain interval at 5.0 Hz and 100% of rest motor threshold (RMT) (total 1,000 pulses). The RMT was defined as the minimal output of stimulation that could evoke a muscle twitch of the right first dorsal interosseous or elicited a motor evoked potential of amplitude of at least 50 *μ*V in at least half of 10 consecutive stimuli recorded by electromyography. According to a previous study exploring the optimal site for localization of DLPFC, the stimulation site was determined by measuring 5.0 cm forward from the point of maximal abductor pollicis brevis stimulation in the parasagittal plane [33]. Numerous studies on the effect of SD on working memory, which used the *N*-back task, have found decreased activation mainly in the frontal lobe [27]. Studies with PET also have observed lower metabolism in prefrontal and parietal areas due to lack of sleep [34]. It has been reported that the frontal-parietal connectivity may possibly be the specific part of the neural circuit of working memory [35, 36]. Therefore, the left DLPFC was selected as the stimulation site. All of the rTMS stimulation was performed by using MagPro R30 (MagVenture, Lucernemarken, Denmark) through a 70.0 mm butterfly-shape coil with the handle posterior and oriented sagittally from 7:40 AM to 8:00 AM on the third day.

### 2.4. Imaging Acquisition

Subjects were scanned three times: first, after a normal night of sleep (8:00 AM to 8:30 AM on the second day); second, after 24 hours of sleep deprivation (7:00 AM to 7:20 AM on the third day); and third, after a session of rTMS stimulation (8:00 AM to 8:20 AM on the third day) with a GE Signa HDxt 1.5 T scanner (General Electric Medical System, Milwaukee, WI, USA). Resting state functional magnetic resonance imaging (rs-fMRI) was performed by using echoplanar imaging gradient echo sequence (EPI) with the following parameters: TR/TE = 2, 000/40 ms, flip angle = 90°, field of view = 240.0 × 240.0 mm^2^, matrix = 64 × 64, and voxel sizes = 3.75 × 3.75 × 5.0 mm^3^. A total of 140 volumes consisting of 32 slices for each were acquired continuously. During the scan after a normal night of sleep, all subjects also underwent a high-resolution T1-weighted anatomical scan (3D-SPGR, 124 slices, TR/TE = 9.1/2.9 ms, flip angle = 20°, field of view = 240.0 × 240.0 mm^2^, matrix = 256 × 256, and voxel sizes = 0.94 × 0.94 × 1.2 mm^3^). During the rs-fMRI imaging data acquisition, subjects were instructed to keep awake, relax with their eyes closed, and remain motionless as much as possible.

### 2.5. Working Memory Test

Every time after undergoing the MRI scanning, the scan was followed by working memory tests which included a short memorized test of letters and numbers during which subjects needed to judge whether a letter/number on the computer screen was contained in the previously appeared sequence of letters/numbers. Each block of the memory test consisted of a three-second viewing of a letter/number sequence, followed by a three-second delay (blank screen), and then a three-second viewing and response time. The letter/number sequence with one, three, or six letters/numbers was randomized to present on the screen and needed to be memorized by the subjects. After a viewing period of blank screen to retain the memory, the subjects were subsequently instructed to respond whether or not the probe letter/number presented on the screen appeared in the previous set of letters/numbers. The response was achieved by using two hand pads equipped with response button to respond YES or NO with their left or right thumbs by memory as accurately and as quickly as possible. The entire task of letter/number recall lasted for seven minutes 20 seconds and contained 48 blocks in total. After finishing the memory test, the reaction times, the time from the beginning of the presentation of the test letter/number to the button response, and the correct number of response (response accuracy) were recorded for every block. All subjects were not trained before taking the working memory test, and they were only notified about the design and the rules.

### 2.6. Image Processing

Prior to preprocessing, the first five volumes of fMRI datasets of each participant were discarded to eliminate the magnetization equilibrium effects and allow the participants to adapt the circumstances. The preprocessing and statistical analysis of the remaining fMRI images were performed by using Statistical Parametric Mapping software package (SPM8, http://www.fil.ion.ucl.ac.uk/spm), with the following steps: (1) correct the time delay between slices during acquisition, (2) realign the images to the first volume to correct the spatial head motion and exclude the fMRI datasets if the motion was more than 3.0 mm of translation or greater than 1° of rotation in any direction, (3) normalize the realigned images to the stand space of the Montreal Neurological Institute (MNI) and resample with a voxel size of 3.0 × 3.0 × 3.0 mm^3^, and (4) also smooth normalized images with an isotropic Gaussian kernel of 8.0 mm and high-pass filter with a cutoff frequency at 128 seconds.

Following the spatial preprocessing, the temporal band-pass filter (0.01-0.08 Hz) was performed for the time series of the spontaneous BOLD signal of each voxel to remove the possible effects of low-frequency drift and high-frequency noise. The linear trend was also estimated and removed from the data. Next, the amplitude of low-frequency fluctuation (ALFF) calculation was carried out by using the REST software (http://www.restfmri.net), generating an individual ALFF map and a standardized mean ALFF map which was obtained by dividing by the individual's global mean ALFF value for each voxel. Finally, one-sided one sample *t*-test was performed to characterize where the standardized ALFF value was significantly larger than the one in the brain [37]. Paired *t*-tests were used to observe the spontaneous activity differences and changes between SD and normal sleep state as well as between rTMS treatment and SD. After the one sample *t*-test, the significant brain regions (*p* < 0.05) were obtained and used as a mask during the paired *t*-test to increase the reliability of the results.

### 2.7. Statistical Analysis

Response accuracy and reaction time were utilized to evaluate the working memory ability and changes in cognitive function associated with SD and rTMS treatment. The mean and standard deviation of the response accuracy and reaction time were calculated for each test and each group. A one-way ANOVA and multiple comparisons were carried out to determine differences of the same assessment of three states and between every two states. Statistical analysis was performed by using the IBM SPSS 22.0 software package.

### 2.8. Correlation Analysis

To further understand whether behavioral performance and changes of working memory exist corresponding neural activity expression, the Pearson correlation coefficients between memory measurements (response accuracy and reaction time) and ALFF values in the cognition-related brain areas were computed. The ALFF values were extracted from the brain regions that showed significant changes after SD and rTMS. Centering on the peak coordinate of the brain region, the mean ALFF value was calculated from 27 voxels around, and the alteration between SD and normal sleep as well as between SD and after rTMS was all calculated. The changes of both ALFF and performance were obtained by a subtraction of the corresponding values between two states.

## 3. Results

### 3.1. Working Memory Test

**Figure 1:**
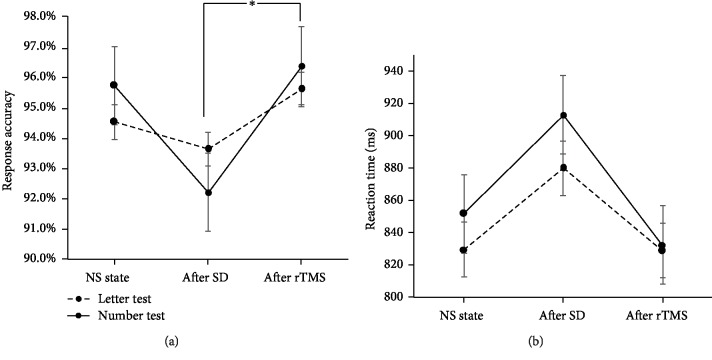
Response accuracy (a) and reaction time (b) of letter and number and spatial position working memory assessment after normal sleep, SD, and rTMS treatment. NS: normal sleep; SD: sleep deprivation; rTMS: repetitive transcranial magnetic stimulation. ∗ represents *p* < 0.05.

Table 1 illustrates the mean and standard deviation of the response accuracy of working memory scores of letter and number tests after normal sleep, SD, and rTMS treatment, respectively.  Figure 1 shows the curve of behavioral performance of three statuses. No significant main effects were identified for response accuracy in letter (*F*_(2, 48)_ = 0.311, *p* = 0.734) and number (*F*_(2, 48)_ = 3.077, *p* = 0.055) and reaction time in letter (*F*_(2, 48)_ = 0.917, *p* = 0.406) and number (*F*_(2, 48)_ = 1.729, *p* = 0.188). After the multiple comparison, we observed a nonsignificant reduction of response accuracy both in letter and number assessments after SD, compared to the normal sleep state (*p* = 0.723; *p* = 0.057). Interestingly, the response accuracies of the letter (*p* = 0.435) and number tests were evidently increased after rTMS treatment in contrast to SD and showed significant changes in the response accuracy of number (*p* = 0.026). In contrast to the response accuracy, reaction time revealed opposite results which showed a rise (letter: *p* = 0.254; number: *p* = 0.237) after SD and reduction (letter: *p* = 0.240; number: *p* = 0.073) after rTMS treatment nearly tending to the reaction time of the normal sleep state. Also, an inverse efficiency score (IES) combining response accuracy and reaction time into a single score was also calculated and analyzed [38]. Similarly, the significant difference was just observed after rTMS relative to the SD status in the number test (*p* = 0.019) after multiple comparison. No significant results were observed both in ANOVA analysis (letter: *F*_(2, 48)_ = 0.818, *p* = 0.447; number: *F*_(2, 48)_ = 3.139, *p* = 0.052) and multiple comparisons (letter, SD vs. RS: 0.311, rTMS vs. SD: 0.246; number, SD vs. RS: 0.091). These findings suggested that rTMS could improve the dulled working memory induced by SD, especially the memory of number.

### 3.2. Dynamic Changes of Neural Activity

**Figure 2:**
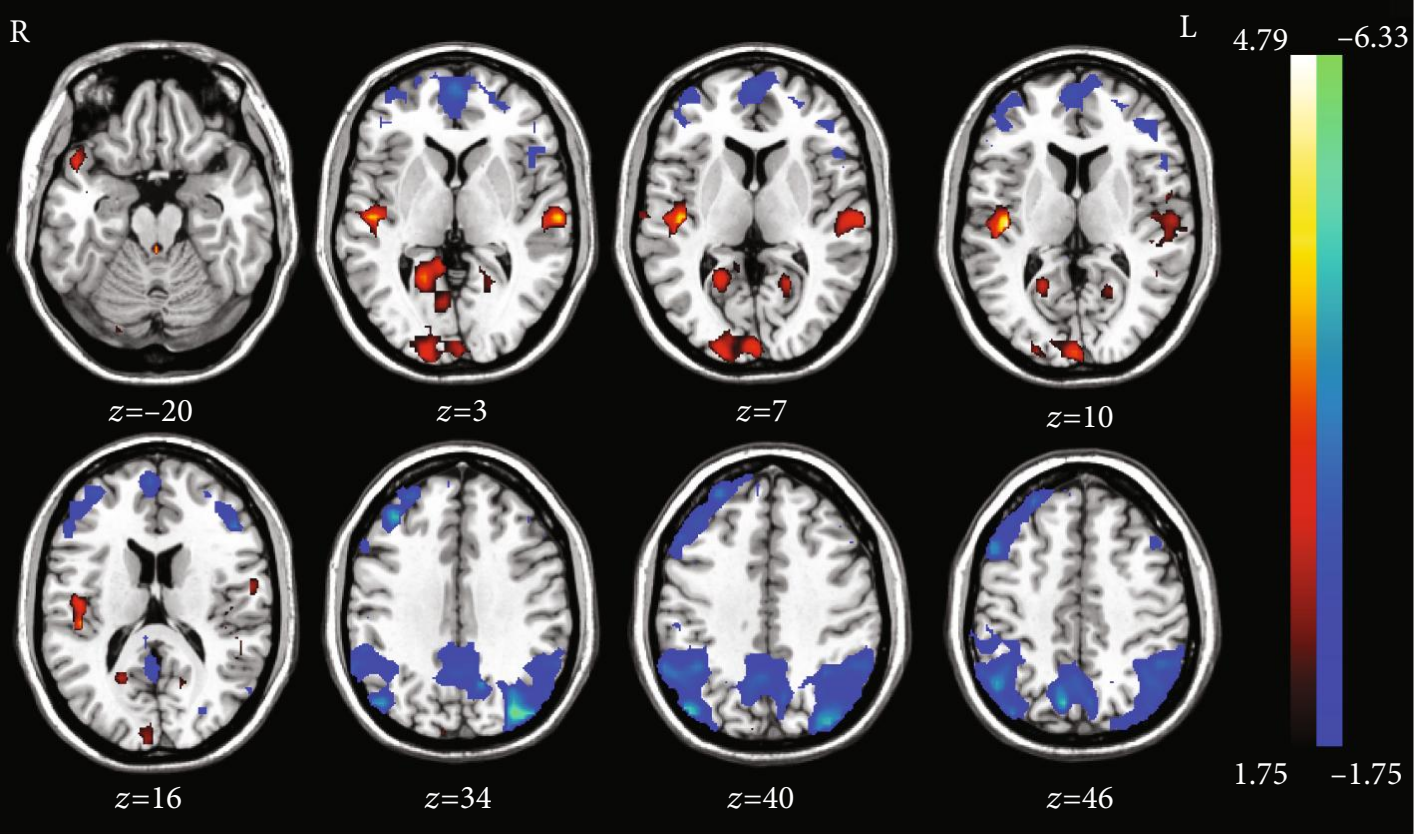
Comparison of the ALFF between SD and NS. The decreased spontaneous neural activity (blue color) after SD mainly located in the default mode network-related brain regions including the posterior cingulate cortex, precuneus, angular gyrus, medial prefrontal cortex, and inferior parietal cortex (*p* < 0.05, Alphasim-corrected).

**Figure 3:**
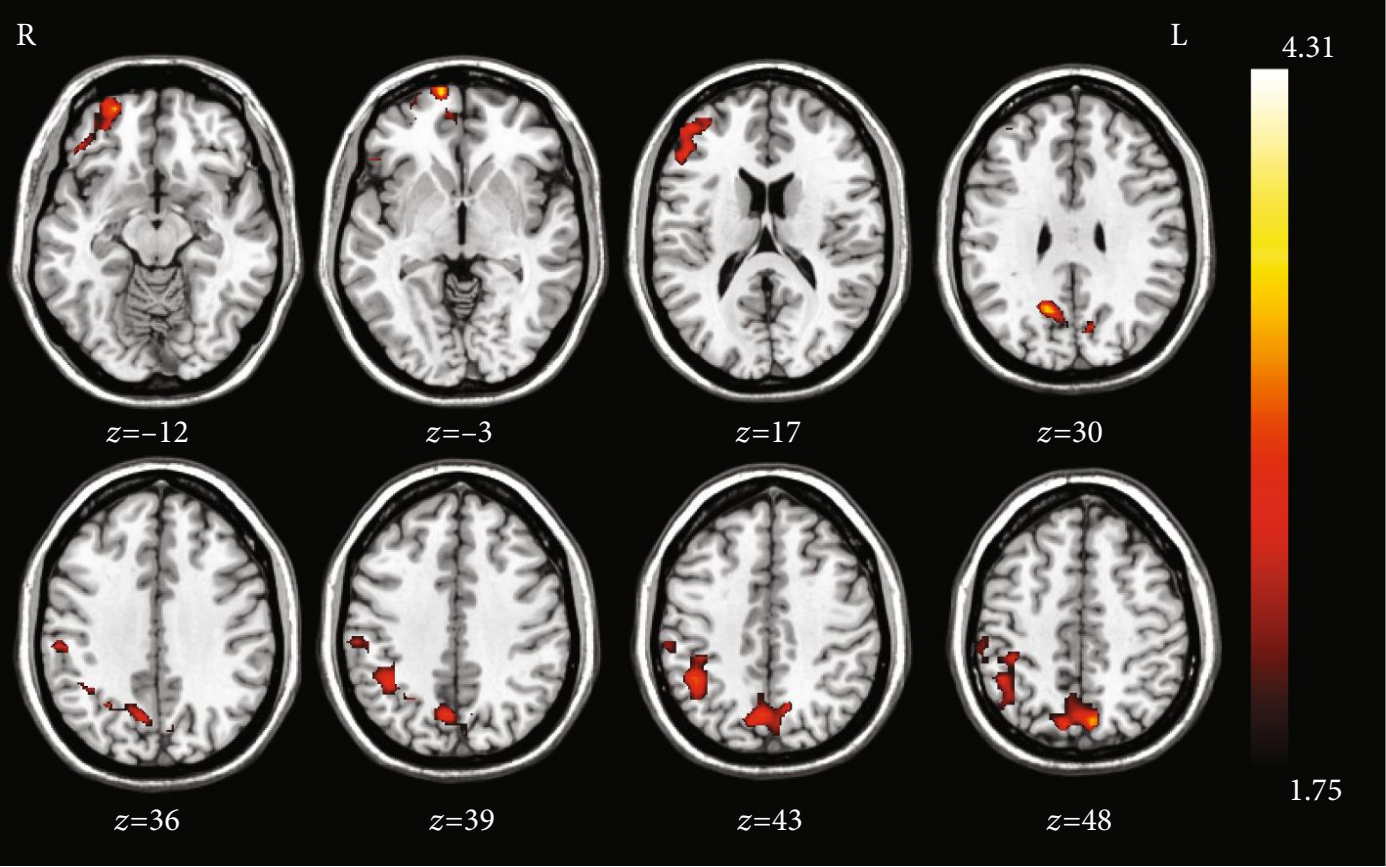
Comparison of the ALFF between rTMS treatment and SD. The neural activity-decreased brain regions (bilateral precuneus, right angular gyrus, and right middle prefrontal cortex) after SD showed a significant increase (red color) after rTMS treatment (*p* < 0.05, Alphasim-corrected).

ALFF could reflect the intensity and brain areas of the spontaneous neural activity. In this study, the ALFF after the normal sleep, SD, and rTMS treatment were all obtained and analyzed to identify the SD and rTMS effects on neural activities of cognition-related brain regions. Compared with the normal sleep state, significantly decreased ALFF values were observed in the bilateral precuneus, angular gyri, parietal cortices, middle frontal cortices, left inferior frontal cortex, and left superior frontal cortex while significantly increased ALFF values were detected in the bilateral superior temporal cortex, left middle temporal cortex, right inferior occipital cortex, right insula, and bilateral calcarine lobe after SD (Table 2, Figure 2). To further clarify the influence and modulation on the neural system of rTMS, the comparison of ALFF between SD and rTMS treatment state was conducted. We detected that bilateral precuneus, right angular gyrus, right inferior parietal lobe, right supramarginal gyrus, right inferior frontal cortex, right middle frontal cortex, and right superior frontal cortex were significantly increased after rTMS which revealed opposite changes after SD (Table 3, Figure 3). Obviously, these regions showing significant changes after SD and rTMS were mainly comprised in the default mode network (DMN) and other cognition-related brain regions. Taken together, these findings may further support the cognitive behavioral results that rTMS could regulate the working memory decline induced by SD.

### 3.3. Correlation between ALFF and Behavioral Data

**Figure 4:**
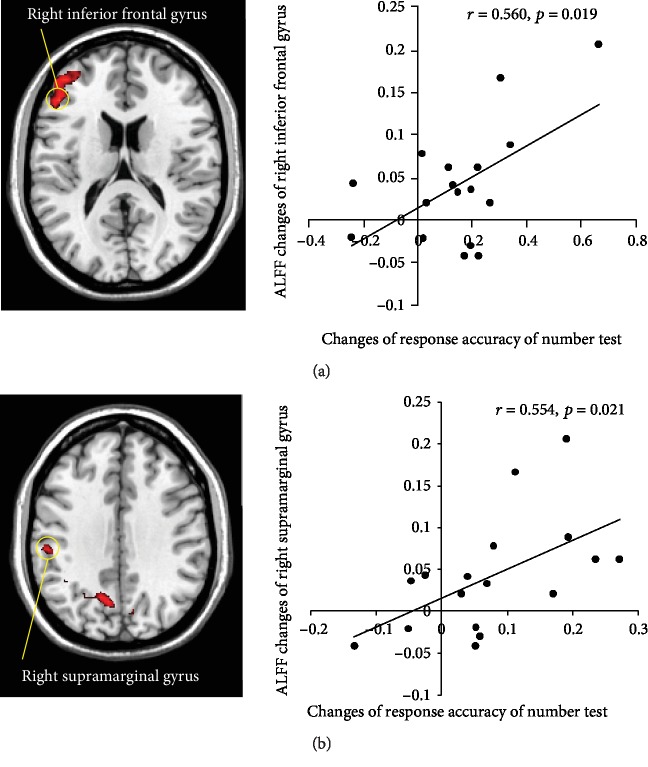
Correlation between the changes of response accuracy and ALFF values. Left view of the figure shows the comparison results of ALFF between rTMS and SD in the right inferior frontal gyrus (a) and right supramarginal gyrus (b). Right view of the figure exhibits the correlation results.

The correlation coefficient is always used to reflect the relationship between distinct evaluation indicators, especially between behavioral measurements and physiological indexes from medical imaging, and molecular biology. In this study, the correlation analysis was conducted to understand the relationship and consistency between working memory ability changes of letter and number and the ALFF changes of related brain regions. Finally, we observed that changes of response accuracy of the number test were significantly positively correlated with the changes of ALFF values of the right supramarginal gyrus (*r* = 0.554, *p* = 0.021) and the right inferior frontal gyrus (*r* = 0.560, *p* = 0.019) after rTMS treatment relative to SD (Figure 4). These findings possibly suggest that the neural activity changes of these regions could predict recovery of the memory of number after rTMS treatment.

## 4. Discussion

This study is aimed at exploring the effects of rTMS, which is a potent acting neurological/psychiatry intervention, on the working memory impairment induced by SD. After 24 hours of SD, we observed decreased response accuracy and increased reaction time on the working memory test of number and letter after SD and observed significantly increased response accuracy of number after rTMS. Consistent with the behavioral performance, we found that the spontaneous neural activities of the cognition-related brain regions comprising the precuneus, frontal gyrus, and angular gyrus, as well as the parietal lobe, were reduced after SD and were significantly increased after rTMS. In addition, the changes of response accuracy of number showed a significantly positive correlation with neural activity changes of the right inferior frontal gyrus and right supramarginal gyrus.

### 4.1. Working Memory and ALFF Changes after SD

Growing evidence has demonstrated the critical impairments of SD on various cognitive abilities, especially on working memory both in younger and older healthy adults, after total SD or partial SD [14, 15, 39–43]. Behavioral results of almost all these past studies found that subjects showed a significantly lower response accuracy rate, longer reaction time, and higher lapse rate on the working memory test after SD relative to the normal sleep state or rested waking state. Our findings are consistent with these studies and show a decreased response accuracy and increased reaction time in both number and letter working memory tests even though these effects were not significant. Furthermore, for total SD, the worst negative effects were not at the end of the total SD but during the mid-time point in which data of the working memory test were collected at three time points (10:00 PM, 04:00 AM, and 06:00 AM). The slowest reaction time of the working memory task and the weakest cerebra response were actually detected during the early morning (04:00 AM) [14]. However, at the end of the SD period (6:00 AM), the reaction time was still significantly slower than that before SD which indicates the significant damage on working memory after SD in self-controlled studies. Also, several studies of 24-hour SD also detected significantly impaired working memory at about 6:00 AM or 7:00 AM [10, 44], which is consistent with the time of the memory test in our study. These all can prove the existence of the effect of SD, with or without a sham rTMS condition in the study. In addition, specifically for emotional working memory, one night of SD may just impair the accuracy but not reaction time [41]. This similar performance was observed after rTMS in our study and just showed significant change in the response accuracy of the number test but not reaction time.

Neuroimaging studies also have been conducted to assess the corresponding cerebral response and their relationship with behavioral changes in previous studies. In these studies, the frontal gyrus and parietal lobe showed more activation changes during the cognitive test, including working memory, verbal learning, thinking fluency, and attention test [12, 15, 45, 46]. Except for these two brain regions, studies, which used resting state fMRI, also showed decreased ALFF in the precuneus, angular gyrus, and thalamus [11, 32, 43]. In addition, SD disturbed functional connectivity and long/short range of functional connectivity density in the precuneus, posterior cingulate cortex, parietal lobe, and prefrontal cortex [32, 43, 47]. Nearly almost all of these brain areas, which showed abnormal activation, spontaneous activity, and functional connectivity, were observed in our study as well. Therefore, the impairment of behavioral performance after SD could be actually detected by using neuroimaging methods. Also, this evidence from functional neuroimaging was further proved in a structural imaging study which observed a thinned cortical thickness precuneus and posterior cingulate cortex after SD [48].

### 4.2. Recovered Working Memory Ability and ALFF Values after rTMS

We observed that both the behavioral performance and spontaneous neural activity were recovered after rTMS which showed and increased response accuracy, reduced reaction time, and increased ALFF values of cognition-related brain regions in our study.

rTMS is gradually recognized as a useful neural modulatory tool in cognitive neuroscience which can modulate the brain activity and brain function. Animal studies of rTMS on young Octodon degus reported that both one and several sessions of high-frequency rTMS were able to significantly improve the impaired alteration of learning and working memory functions induced by SD without any side effects [28, 29]. Another human rTMS study observed the similar results in which 5.0 Hz rTMS was applied to the left occipital cortex while participants performed a working memory task after two days of SD. This study found that similarly to non-SD subjects, participants receiving active rTMS did not show slowing and lapsing performance compared to subjects who received sham rTMS and which exhibited degraded performance in the task [31]. These results may indicate that rTMS applied concurrently with the working memory task could prevent the development of working memory impairments caused by SD and affect the neural circuitry in which increased activation during SD was observed in the left middle occipital gyrus. However, the design of this study was different from ours. rTMS sessions were performed during the SD period which may disturb the brain activation obtained at the end of SD. Separate fMRI acquisition after SD and rTMS as well as the comparison of brain activation between could better reveal the modulation of rTMS on neural activity. This approach was applied in our study in which rTMS was not applied simultaneously with the working memory test but also showed significantly improved performances. The correlation was conducted between rTMS-induced ALFF changes and working memory performance changes after rTMS could intuitively reflect the relationship between behavioral performance and neural activity after rTMS. This may indicate that except for preventing the impairment, rTMS also could remedy or at least ameliorate the impairment in cognition caused by SD. Furthermore, the subjects were not trained before participating in the memory test. During the test, the pictures with number or letter randomly appeared. The learning effect resulting from practice could be eliminated. Therefore, the results indicate that rTMS could retrieve the cognitive impairment by modulating the regional brain activity of cognition-related brain areas.

Interestingly, the ALFF value increases were mainly observed in the frontal gyrus, precuneus, angular gyrus, parietal lobe, and supramarginal gyrus after rTMS which were decreased after SD. The supramarginal gyrus has a well-established role in the language and verbal working memory [49–51] and especially in memory retrieval showing stronger interaction with the hippocampus [52]. The other four regions are the main component of the DMN network which has been proved very important for complex cognition and working memory and were associated with sleep quality [53, 54]. Particularly angular gyrus and one hub of DMN have been proven as a great contribution to working memory and have a potential involvement in the global integration of information [50, 55]. The essential role of the frontal gyrus and parietal lobe for working memory is a fact that has been confirmed in many previous studies [56–58]. Furthermore, the parietal-frontal connectivity may be the specific part of the neural circuit of working memory [35, 36]. Therefore, by modulating the activity of the brain regions in the neural circuit of working memory, high-frequency rTMS over the frontal cortex could realize the improvement of working memory ability after SD. Further functional connectivity research with large sample size is needed to elucidate the underlying mechanism on modulating the neural circuit of rTMS.

### 4.3. Correlation between Response Accuracy and ALFF Values

It is noteworthy to emphasize the positive correlation between the change of response accuracy and the ALFF changes of the inferior frontal gyrus and the supramarginal gyrus. We have introduced the importance of the frontal gyrus and supramarginal gyrus in the above discussion. Also, one previous study reported that the prefrontal gyrus was most sensitive to sleep loss [59]. Therefore, it showed more increased spontaneous activity after rTMS and may be more sensitive to the treatment. Identical to the frontal gyrus, the supramarginal gyrus may be more influenced by SD and easier activated by neural stimulation treatment which needs to be clarified in the future studies.

## 5. Limitations

There are some limitations to this study. First, a relatively small sample size of participants was recruited in this study to characterize the modulation of rTMS on working memory behavior and spontaneous neural activity. This limitation may result in the moderate nonsignificant impact of SD on behavioral performance in our study. This needs to be further verified in the future studies with a large sample size. Second, significantly more female subjects were enrolled in our study than male subjects. Third, a self-controlled trial was adopted in this study with comparison between pre- and poststate of the same group participants. It would be better if a control group with sham rTMS stimulation with age-, gender-, and education-matched participants was included in this study. In future studies, it would be vital to verify our results with a large sample size and good-designed trials. Fourth, in this study, ALFF was employed to estimate the spontaneous activity changes induced by SD and rTMS. The ALFF approach showed higher sensitivity and specificity in detecting spontaneous brain activity and distinguishes some diseases from the normal control than the other fMRI indices [60, 61]. However, network analysis including functional connectivity and effective functional connectivity analysis could better reflect the path of modulation of rTMS on working memory. In future studies, it is worthwhile to investigate the effect on neural circuit of rTMS and the relationship with corresponding behavioral performance in order to better understand the neural mechanism of rTMS on working memory.

## 6. Conclusion

In conclusion, this study suggests that the impairment of working memory induced by SD could be rescued by rTMS. The improvement of behavioral performance may be attributed to the positive modulation of rTMS on the spontaneous neural activity of cognition-related brain areas.

## Figures and Tables

**Table 1:** Working memory test results of three states.

States	Letter test		Number test	
	RA	RT (ms)	RA	RT (ms)
RS	0.945 ± 0.062	839.34 ± 130.08	0.957 ± 0.056	867.20 ± 123.12
SD	0.936 ± 0.069	892.76 ± 154.40	0.922 ± 0.068	920.88 ± 150.73
rTMS	0.956 ± 0.086	837.68 ± 117.55	0.964 ± 0.024∗	838.74 ± 115.93

RA: response accuracy; RT: reaction time; RS: normal sleep; SD: sleep deprivation; rTMS: repetitive transcranial magnetic stimulation. ∗ represents a significant change between the state of SD and rTMS (*p* < 0.05).

**Table 2:** Regions of significantly changed neural activity (ALFF) following sleep deprivation compared with activity following normal sleep.

Condition	Brain regions of peak voxel	L/R	Voxels	*T* value of peak voxel	MNI coordinate of peak voxel
SD < NS	Precuneus	L	380	-4.50	-15, -64, 34
	Precuneus	R	401	-5.42	9, -73, 43
	Angular gyrus	L	268	-3.82	-39, -61, 46
	Angular gyrus	R	327	-5.46	48, -64, 49
	Parietal lobe	L	289	-3.70	-57, -49, 40
	Parietal lobe	R	280	-3.98	42, -52, 40
	Occipital lobe	L	244	-6.33	-33, -82, 31
	Occipital lobe	R	79	-4.60	48, -76, 31
	Middle frontal gyrus	L	318	-3.69	-39, 17, 49
	Middle frontal gyrus	R	605	-5.07	39, 35, 31
	Inferior frontal gyrus	L	212	-4.23	-48, 29, 16
	Superior frontal gyrus	R	143	-3.60	3, 56, 13
SD > NS	Middle temporal gyrus	L	62	3.99	-63, -19, 1
	Superior temporal gyrus	L	131	2.90	-42, -31, 10
	Superior temporal gyrus	R	249	4.38	48, -19, -2
	Insula lobe	R	72	2.38	48, -1, -2
	Lingual gyrus	R	317	3.45	18, -55, 1
	Calcarine gyrus	L	184	3.47	0, -97, 7
	Calcarine gyrus	R	156	3.31	18, -100, 4

Every result was significant at a minimum *p* value of 0.05 with Alphasim correction, cluster size of more than 213 voxels. L: left hemisphere; R: right hemisphere; SD: sleep deprivation; NS: normal sleep.

**Table 3:** Regions of significantly changed neural activity (ALFF) following rTMS treatment.

Condition	Brain regions of peak voxel	L/R	Voxels	*T* value of peak voxel	MNI coordinate of peak voxel
rTMS > SD	Precuneus	L	90	3.34	-6, -76, 46
	Precuneus	R	195	3.48	15, -64, 28
	Angular gyrus	R	64	2.39	48, -67, 49
	Parietal lobe	R	132	3.07	42, -49, 40
	Supra marginal gyrus	R	89	2.99	54, -28, 31
	Inferior frontal gyrus	R	103	3.39	48, 29, 13
	Middle frontal gyrus	R	244	3.49	24, 56, -14
	Superior frontal gyrus	R	42	4.31	12, 65, 1

Every result was significant at a minimum *p* value of 0.05 with Alphasim correction, cluster size of more than 219 voxels. L: left hemisphere; R: right hemisphere; SD: sleep deprivation.

## Data Availability

The xlsx behavioral data used to support the findings of this study and data regarding correlation analyses and the results are included within the supplementary information file. The data of fMRI used to support the findings of this study have not been made available because of the large number of original image files.

## Supplementary Materials

Supplementary Materials The xlsx behavioral data used to support the findings of this study are included within the supplementary information file.
